# Identification and characterization of *Bacillus thuringiensis* and other *Bacillus cereus* group isolates from spinach by whole genome sequencing

**DOI:** 10.3389/fmicb.2022.1030921

**Published:** 2022-11-30

**Authors:** Xingchen Zhao, Athanasios Zervas, Marc Hendriks, Andreja Rajkovic, Leo van Overbeek, Niels Bohse Hendriksen, Mieke Uyttendaele

**Affiliations:** ^1^Food Microbiology and Food Preservation Research Unit, Department of Food Technology, Safety and Health, Faculty of Bioscience Engineering, Ghent University, Ghent, Belgium; ^2^Department of Environmental Science, Aarhus University, Roskilde, Denmark; ^3^Wageningen Plant Research, Wageningen University and Research, Wageningen, Netherlands

**Keywords:** *Bacillus thuringiensis*, biopesticide, WGS, qPCR, microbial source tracking

## Abstract

*Bacillus thuringiensis* (Bt), used as a biological control agent (BCA), can persist on plants, and from there can be introduced into the final food product. In routine food safety diagnostics, these Bt residues cannot be distinguished from natural populations of *Bacillus cereus* present in plants and all are enumerated as “presumptive *B. cereus*.” In this study, information on eventual use of Bt biopesticides, brand, application times and intervals provided by three food processing companies in Belgium, were integrated with quantitative data on presumptive *B. cereus* measured from fresh to frozen food products. This information together with data on genomic similarity obtained *via* whole genome sequencing (WGS) and *cry* gene profiling using a quantitative real-time PCR (qPCR) assay, confirmed that six out of 11 Bt isolates originated from the applied Bt biocontrol products. These identified Bt strains were shown to carry enterotoxin genes (*nhe, hbl, cytK-2*) and express Hbl enterotoxin *in vitro*. It was also noted that these Bt biopesticide strains showed no growth at standard refrigeration temperatures and a low or moderate biofilm-forming ability and cytotoxic activity. Our results also showed that the use of Bt as a BCA on spinach plants in the field led to higher residual counts of Bt in spinach (fresh or frozen) in the food supply chain, but the residual counts exceeding at present commonly assumed safety limit of 10^5^ CFU/g was only found in one fresh spinach sample. It is therefore recommended to establish a pre-harvest interval for Bt biopesticide application in the field to lower the likelihood of noncompliance to the generic *B. cereus* safety limit. Furthermore, WGS was found to be the best way to identify Bt biopesticide isolates at the strain level for foodborne outbreaks and clinical surveillance. The developed qPCR assay for screening on the presence of *cry* genes in presumptive *B. cereus* can be applied as a rapid routine test as an amendment to the already existing test on Bt crystal proteins determined *via* phase-contrast microscopy.

## Introduction

*Bacillus cereus* group (*Bacillus cereus sensu lato*) strains are ubiquitous in the environment, and those prevalent in the soil can colonize both the rhizosphere and the phyllosphere of plants (Stenfors Arnesen et al., 2008; Ehling-Schulz et al., 2019). *Bacillus thuringiensis* (Bt), a member of the *B. cereus* group, has been used as an effective biological control agent (BCA) for decades to control insects in agriculture and has the majority of the market share of the commercially produced microbial BCAs (Lacey et al., 2015). Bt used as BCAs can reach plants in quite high numbers (10^5^–10^6^ CFU/g; De Bock et al., 2021). They can persist on crops and can be consequently found on the final products throughout the food supply chain (De Bock et al., 2019; Biggel et al., 2022b). Members of the *B. cereus* group are estimated to be attributed to 1.4%–12% of foodborne outbreaks worldwide (Grutsch et al., 2018). *Bacillus cereus* group is an etiological agent of two types of foodborne diseases: food intoxication and toxico-infection (Jovanovic et al., 2021). The former is caused by the intake of a food product that contains the emetic toxin cereulide, a toxin produced by the emetic members of the *B. cereus* group; typical symptoms include nausea and vomiting (Ehling-Schulz et al., 2015; Rouzeau-Szynalski et al., 2020). The latter is caused by the consumption of food products containing high numbers (>10^5^ CFU/g) of *B. cereus* vegetative cells or spores, which subsequently produce one or multiple enterotoxins in the small intestine; typical symptoms include abdominal pain and diarrhea (Dietrich et al., 2021; Jovanovic et al., 2021). *Bacillus cereus sensu stricto* (according to the taxonomic framework in Genome Taxonomy Database; GTDB) is not the only causative agent of toxico-infection, as it has been reported that other members of the *B. cereus* group are also associated with toxico-infection illnesses and outbreaks (Lund et al., 2000; Guinebretière et al., 2013; Bonis et al., 2021).

In routine food safety diagnostics, Mannitol Egg Yolk Polymyxin (MYP) agar is often used as a selective medium for the identification and enumeration of presumptive *B. cereus* according to International Organization for Standardization (ISO) 7932 (ISO, 2020). However, this approach cannot differentiate among different species in the *B. cereus* group, except for *B. mycoides* and *B. pseudomycoides* (GTDB species) with their distinctive rhizoid growth, and as such, all other species are identified as either *B. cereus* or presumptive *B. cereus* (EFSA, 2016; De Bock et al., 2021). Since 2020, ISO has recommended the use of phase-contrast microscopy to identify parasporal crystals associated with Bt (ISO, 2020); but irregular crystals might be confused by other internal bodies, rendering this discrimination method unreliable in certain cases (EFSA, 2016). Due to the high genetic similarity between species in the *B. cereus* group, the traditional Matrix-assisted laser desorption/ionization time-of-flight mass spectrometry (MALDI-TOF MS) fingerprinting method is also limited in providing discrimination at the species level (De Bock et al., 2021). Hitherto no rapid, high throughput and reliable method in routine food safety testing are available to differentiate Bt from *B. cereus* and among *B. cereus* group strains at the species level (EFSA, 2016). Therefore, the reported outbreaks related to Bt and other *B. cereus* group members might be underreported or even overlooked. Currently, the only way to unambiguously assign isolates to a specific species within the *B. cereus* group is through whole-genome sequencing (WGS; EFSA, 2016).

Applying WGS as a new method has important applications in food safety, particularly for foodborne disease surveillance and outbreak investigations (Ronholm et al., 2017). Thus, WGS of *B. cereus* group isolates has started to be used for food safety surveillance (Miller et al., 2018; Carroll et al., 2019). Very recently, it has been pointed out that Bt was associated with foodborne outbreaks, and the implicated Bt isolates were genetically similar to Bt strains used in commercially authorized BCA products (EFSA, 2016; Johler et al., 2018; Bonis et al., 2021; Biggel et al., 2022a). This raises concerns regarding potential food safety risks related to the use of Bt as a BCA for edible plants. However, the available data are insufficient to assess the actual risks of Bt for food safety. Data gaps include agro-technical aspects related to the use of Bt biopesticides, epidemiological evidence from clinical and food samples, as well as possible cross-contamination scenarios. As such, the actual risk assessment of Bt biopesticide strains to humans originating from the food supply chain is still debatable (EFSA, 2016; Raymond and Federici, 2017; De Bock et al., 2021). In this study, we focus on spinach as one of the horticultural products frequently treated with Bt-based BCA. For this purpose, we cooperated with three spinach-processing companies in Belgium, which provided spinach samples, and information on their use of Bt biopesticides including the brand of used Bt biopesticides, the application times and intervals. To assess the potential exposure of Bt biopesticides to consumers by the end product (fresh or frozen spinach) and the hazard of Bt biopesticide strains, data on the occurrence of presumptive *B. cereus* were combined with phenotypic, genetic and cytotoxic characterizations of spinach isolates. Further, WGS and a quantitative real-time PCR (qPCR) assay developed by Wageningen University & Research (WUR) were used to investigate whether the recovered Bt isolates from Bt-treated spinach samples were the same as the strains present in the used Bt biopesticides.

## Materials and methods

### Bacterial strains, cell lines, and culture conditions

The six *B. cereus* group reference strains are listed in Table 1. Isolates from Bt biopesticides acted as reference strains for the identification of Bt biopesticide strains. Bw 472, a psychrotolerant strain (Zhao et al., 2021), was used as the positive control for the ability to grow at low temperatures. Bc 836 was used as the positive control for the cytotoxicity assay and the biofilm formation capacity, as it has been reported as a cytotoxic and biofilm-forming strain (Kilcullen et al., 2016; Kwon et al., 2017). All strains were stored in 15% glycerol stocks at −75°C and cultured in 9 ml of brain heart infusion broth (BHI) at 30°C. The Caco-2 cell line (HTB-37^™^) was obtained from the American Type Culture Collection and cultured in Dulbecco’s Modified Eagle’s Medium (DMEM) with high glucose (GlutaMAX^™^ Supplement, pyruvate; Gibco, United States) supplemented with 10% fetal bovine serum (FBS) and 1% non-essential amino acids (NEAA) in a humidified incubator with 10% CO_2_ at 37°C. Cells were sub-cultured with 0.25% trypsin–EDTA when reaching 80%–90% confluency.

**Table 1:** Reference strains used in this study.

Species	LFMFP number	Collection number	Origin	Genome accession number in NCBI
*Bacillus thuringiensis*	xentari	ABTS-1857	XenTari^®^ WG^#^	GCA_025210065.1
	turex^$^	GC-91	Turex^®^ WG	–
	dipel	ABTS-351	Dipel^®^ WG^#^	GCA_025210105.1
	delfin	SA-11	Delfin^®^ WG^*^	GCA_025209995.1
*Bacillus weihenstephanensis*	472	LMG 18989	Pasterurized milk	–
*Bacillus cereus*	836	ATCC 14579	Type strain	–

# Kindly provided by Valent BIoSciences LCC (Libertyville, IL, United States).

$ Kindly provided by Aarhus University, it is the isolate from Turex^®^ WG collected and stored in 1995, which is the same strain used in the commercial product Agree^®^ 50 WG.

* Kindly provided by Biobest (Westerlo, Belgium).

### Prevalence study of *Bacillus cereus* group from fresh to frozen spinach

#### Sample collection

Samples of fresh spinach leaves taken directly from the field (Company 1, 2), after washing (Company 1), and frozen (Company 1, 2, 3) spinach samples in clean plastic bags were provided by three food processing companies in Belgium from June to July 2020. Registration forms with sampling information (i.e., treated with Bt biopesticides or not in the field and application times) were asked to be filled out by the companies (summarized sampling information is shown in Supplementary Table 1). The collected fresh and frozen samples were stored at <8°C and at −20°C, respectively. They were transported in boxes with cooling elements to the laboratory at Ghent University (UGent) within 48 h, along with the completed registration forms. The samples were immediately stored at appropriate temperatures (4°C or −20°C) and analyzed within 24 h at UGent.

#### Enumeration and isolation of *Bacillus cereus* group

For each sample, 25 g were weighed in sterile stomacher bags, mixed with 225 ml peptone physiological solution [PPS; 1 g/L peptone (Oxoid, United Kingdom) + 8.5 g/L NaCl (Sigma-Aldrich, United States)], and homogenized by a stomacher (Lab Blender 400, United Kingdom) for 1 min. The homogenate was 10-fold serially diluted in PPS, and 0.1 ml of each dilution was spread-plated on MYP (Oxoid) agar plates in duplicates and incubated at 30°C for 24 h. After incubation, typical colonies of presumptive *B. cereus* were identified and counted. Three to five *B. cereus* colonies with different morphologies (if any) were selected and streaked to purity on MYP agar plates. Isolates, resulting from single colonies, were stored in BHI broth with 20% glycerol at −75°C for further analyses.

### Identification of presumptive Bt by phase-contrast microscopy

Overnight cultures (100 μl) of the presumptive *B. cereus* isolates were inoculated on strengthened Nutrient Agar (sNA; 28 g/L Nutrient Agar (Oxoid) + 0.04 g/L MgCl_2_ (Sigma-Aldrich) + 0.10 g/L CaCl_2_ (Sigma-Aldrich)) at 30°C for 24–48 h until sporulation was observed by phase-contrast microscopy (ZEISS Axioscope 5, Germany). Isolates with parasporal crystals (a minimum size: approximately 1/4 to 1/2 of the spore) were tentatively identified as presumptive Bt (ISO 7932:2004/Amd 1:2020).

### *Bacillus cereus* group isolate selection and DNA preparation

In total, 34 *B. cereus* group isolates were selected based on the results of the enumeration and the identification of presumptive Bt along with the information on the use of the Bt biopesticides or not. At least one isolate was selected from each presumptive *B. cereus* positive sample. Of the 34 isolates, 21 were identified as presumptive Bt based on the presence of parasporal crystals, of which 12 presumptive Bt isolates were from Bt-treated spinach samples, while the remaining 13 isolates were not identified as presumptive Bt.

DNA from the isolates was extracted from overnight cultures grown in BHI tubes at 30°C using DNeasy^®^ UltraClean^®^ Microbial Kit (QIAGEN, Germany) at UGent. Two batches of DNA were extracted on different days following the manufacturers’ instructions; one batch was sent to Aarhus University (AU) for WGS and the other batch was sent to WUR for the screening of *cry* genes by qPCR analysis. WGS and qPCR were done without previous knowledge about the identity of the isolates.

### Whole-genome sequencing and bioinformatics analyses

DNA was quantified using Qubit 4 (Life Technologies, United States), and libraries were prepared using the Nextera XT sample preparation kit (Illumina, United Kingdom). Sequencing was performed on the Illumina Nextseq 500 platform with the 300 cycle chemistry (2 × 151 pair-end reads). In total, 4 Bt biopesticide strains shown in Table 1 and 34 spinach isolates were sequenced. Low-quality base and Illumina adapter trimming were performed using Trim Galore version 0.6.5^1^ with default parameter settings, and FastQC version 0.11.9^2^ was used for the quality control. Draft genomes were then assembled *de novo* using SPAdes version 3.15.3 with default parameters (Bankevich et al., 2012). The assemblies were assessed by QUAST version v5.1.0rc1 (Gurevich et al., 2013) and BUSCO version 5.2.2 using the dataset bacilli_odb10 (Simão et al., 2015; Manni et al., 2021). Annotation of the assemblies was performed using PROKKA version 1.13 (Seemann, 2014) under default settings.

Comparison and de-replication of genomes were performed with dRep version 3.2.0 (Olm et al., 2017). For dRep, 95% average nucleotide identity (ANI) was used for species delimitation, and “same” genomes were considered those with similarities >99.9% (secondary_clustering_dendrogram). Genome assemblies were submitted to BTyper 2.3.2 (Carroll et al., 2017) for *in silico* virulence gene detection. BTyper3 (Carroll et al., 2020a,b) was used for multi-locus sequence typing (MLST), *panC* group assignment and Bt toxin-encoding gene identification (Camacho et al., 2009; Cock et al., 2009). In addition, *cry* and *cyt* genes were also checked after annotation performed by PROKKA.

Whole-genome single nucleotide polymorphisms (wgSNPs) were detected by mapping trimmed Illumina paired-end reads of spinach isolates to the assembly of the closest (based on ANI and sampling information) Bt biopesticide isolate using the CFSAN SNP pipeline version 2.2.1 (Davis et al., 2015) with the “run” subcommand and default settings. The assembled genome of xentari, which was assigned to sequence type (ST) 15, and reads of nine related spinach isolates from the same ST were run in a folder. The assembled genome of delfin, which was assigned to ST 8, and reads of two related spinach isolates from the same ST were run in another folder. Reads of the respective reference biopesticide isolate were also included in the pipeline to calculate its SNP distances with spinach isolates.

### Identification of Bt biopesticide strains by qPCR assay

#### qPCR development and validation

According to the different *cry* gene profiles (Table 2) present in seven authorized Bt biopesticide strains belonging to Bt subspecies *aizawai* and *kurstaki*, *cry1Aa*, *cry1Ab*, *cry2Aa*, and *cry1D* were selected for the identification of Bt biopesticide strains. These seven authorized Bt biopesticide strains are used as active substances in commercial biopesticide products, which are most commonly applied for biocontrol treatment in the EU. The sequences of *cry1Aa* (CP004876.1), *cry1Ab* (CP004873.1), *cry2Aa* (CP004876.1), and *cry1D* (HY933637.1) were retrieved from NCBI database using indicated nucleotide accession numbers. These *cry* genes were randomly mapped against the Bt biopesticide strain genomes (sequenced at WUR) with the read mapping tool within the CLC Genomics Workbench Software (version 22.0.1) from Qiagen (Aarhus, Denmark) with the following parameters: match score = 1, mismatch cost = 2, insertion cost = 3, deletion cost = 3, length fraction = 0.5, similarity fraction = 0.8. Primers and hydrolysis probes (Table 3) were designed with the PrimerQuest tool from Integrated DNA Technologies (IDT, Coralville, IA, United States) and were ordered from IDT (Leuven, Belgium). All hydrolysis probes were labeled with a 6-carboxyfluorescein (FAM) dye at the five prime end and double quenched with an internal ZEN Quencher and an Iowa Black^®^ FQ Quencher at the three prime end. The amplicon length differed between qPCR assays: for the *cry1AaAb* gene, the amplicon size is 118 bp; for the *cry2Aa* gene, it is 103 bp, and for the *cry1D* gene, it is 93 bp. Specificity was tested *in silico* by BLAST nucleotide sequence comparisons on the strains described in Supplementary Table 2 and in the NCBI non redundant database using MegaBLAST with default parameters (webserver, BLASTN version 2.13.0+; Boratyn et al., 2013). Synthetic double-stranded DNA fragments (gBlocks; IDT, Coralville, IA, United States) consisting of the amplicon sequence extended on the 5′ and 3′ ends as indicated (Supplementary Table 3) were used to test the sensitivity, robustness and efficiency of the *cry1Aa/Ab*, *cry2Aa*, and *cry1D*-directed qPCR assays.

**Table 2:** Presence of *cry* genes in Bt biopesticide strains confirmed by the qPCR assay.

Species	Subspecies	Strain collection number	Representative products	*cry1Aa*	*cry1Ab*	*cry1D*	*cry2Aa*
*B. thuringiensis*	*kurstaki*	ABTS 351	Dipel^®^	√	√		√
*B. thuringiensis*	*kurstaki*	EG 2348	Lepinox^®^Plus	√	√		√
*B. thuringiensis*	*kurstaki*	SA-11	Delfin^®^ WG	√	√		√
*B. thuringiensis*	*kurstaki*	SA-12	Deliver WG^®^	√	√		√
*B. thuringiensis*	*kurstaki*	PB 54	Belthirul^®^	√	√		√
*B. thuringiensis*	*aizawai*	ABTS 1857	XenTari^®^	√	√	√	
*B. thuringiensis*	*aizawai*	GC-91	Agree^®^ 50 WG			√	

**Table 3:** Primers and hydrolysis probes used in the qPCR assay.

Target	Name	Sequence (5′–3′)	Tm (°C)	GC %	Amplicon (bp)
*cry1AaAb*	cry1Aa/Ab_F	GGTACGTACACTTCTCGTAATC	60	45.5	118
	cry1Aa/Ab_R	TGTCTCTTCGTCCATCTGTA	61	43	
	cry1Aa/Ab_P	FAM/TGACGG/ZEN/AGCCTATGAAAGCAATTCTTCTGT/3IABQkFQ	69	45	
*cry2Aa*	cry2Aa_F	GGAGTTTCATCTGGTCTCATAG	60	45.5	103
	cry2Aa_R	CCAGCCAACTTCTAACAAATG	60	42.9	
	cry2Aa_P	FAM/TGCAGC/ZEN/ACGGTCCTCCCTCCTTTA/3IABkFQ	70	58	
*cry1D*	cry1D_F	ACGTGAAAGGGCATGTAG	60	50	93
	cry1D_R	GAACCTCTTGTGACACTTCT	60	45	
	cry1D_P	FAM/ACAATC/ZEN/ACCGTTCAGTCCTGGTTATCCCA/3IABkFQ	70	48	

#### qPCR method

All qPCR assays were performed on the Quantstudio 12 K Flex (Thermo Fisher Scientific, Waltham, MA, United States). Reactions were performed with 2.0 μl target (2 ng/μl) in a 25 μl volume, including 1× Premix Ex Taq^™^ DNA Polymerase (Perfect Real Time; Takara BIO Europe, Saint-Germain-en-laye, France), 0.125 μl 50× ROX reference Dye II, 300 nM forward and reverse primer and 100 nM hydrolysis probe. Transparent MicroAmp^®^ Optical 96-Well reaction plates (Applied BioSystems, Foster City, CA, United States) combined with MicroAmp^™^ optical adhesive film (Applied BioSystems, CA, United States) were used. The temperature profile consisted of an initial denaturation step of 95°C for 2 min, followed by 40 cycles of 95°C for 15 s and 60°C for 1 min. Results were analyzed with the Quantstudio 12 K Flex Software. Threshold and baseline settings for each run were automatically set by the software. In each plate, a gBlock dilution series of the target (10^6^ to 1 copies) and a negative control (water) were analyzed in parallel.

This qPCR method was performed on colony material, and Ct values below 27 were considered valid, while they were not considered valid if the values were above 32, as it was experienced that the background signal then was too high, and results were inconclusive. Ct values were always confirmed by amplification curves. These curves must be sigmoidal, following the two-fold increase to the maximum amplification plateau level. The curves not following two-fold amplifications and not reaching the maximal amplification plateau level were considered as negative.

### Growth ability at low temperatures

According to the genomic similarity covering all detected STs of these isolates from spinach and the origin of these isolates, 23 isolates together with four Bt biopesticide isolates were selected for further downstream processing steps. Growth of selected isolates was evaluated at two refrigerator temperatures (4 and 7°C) and an abuse storage temperature (10°C). Overnight cultures (100 μl) of the presumptive *B. cereus* isolates in BHI were transferred into 9 ml BHI and also streaked on Tryptic Soy Agar (TSA; Oxoid), and subsequently incubated at 7°C for 14 days. After 14 days, if visible growth was observed in BHI or on TSA, a loopful of broth (BHI), or colony material (TSA) was streaked on MYP agar and incubated at 30°C for 24 h. Identity of colonies was confirmed by morphology on MYP using the inoculated spinach isolate as the reference. The same experiment was repeated at 10°C for the isolates which were unable to grow at 7°C, and at 4°C for the isolates which were able to grow at 7°C. All the experiments were repeated twice independently.

### Biofilm formation assay on polystyrene

Biofilm formation capacity of the selected *B. cereus* group isolates was evaluated on polystyrene as described previously (Wijman et al., 2007) with some modifications. Briefly, 10–20% (v/v) overnight cultures in tryptic soy broth (TSB; Oxoid) were adjusted to 10^6^ CFU/ml by optical density (OD) at 600 nm. Each well of sterile Falcon^®^ 96-well polystyrene microplates (Corning, United States) was filled with 200 μl TSB and inoculated with 10% of the diluted overnight culture (final concentration of 10^5^ CFU/ml). Six wells per column were inoculated with the same isolate, and wells that only contained TSB acted as negative controls. To prevent evaporation during incubation, wells on the four sides of the microplates were filled with sterile distilled water and the microplates were wrapped with parafilm. The microplates were statically incubated at 30°C for 24 h. After incubation, the media was removed from each well by carefully pipetting, and wells were washed thrice with 250 μl PPS. The attached biofilm was stained with 0.1% (w/v) crystal violet (CV) for 30 min, then wells were washed thrice again with 250 μl PPS. Subsequently, 200 μl of 33% glacial acetic acid was added for 30 min to release CV bound by biofilm. Crystal violet, dissolved in 33% glacial acetic acid was transferred into a new 96-well microplate and the absorbance was measured at 595 nm in a VersaMax microplate reader (Molecular Devices, United States). Experiments were done independently on three different days as three biological replicates.

The cut-off value of the optical density (OD_cut_) was calculated using the following formula (Singh et al., 2017):

OD_cut_ = Average OD (OD_avg_) of negative control + 3 × standard deviation (SD) of ODs of negative control.

Biofilm formation capacity was categorized by the following criteria (Singh et al., 2017; Leoney et al., 2020):

(1) Non-biofilm-former: OD ≤ OD_cut_; (2) Weak biofilm-former: OD_cut_ < OD ≤ 2 × OD_cut_; (3) Moderate biofilm-former: 2 × OD_cut_ < OD ≤ 4 × OD_cut_; Strong biofilm-former: OD > 4 × OD_cut_.

### Hemolysin BL enterotoxin detection and cytotoxicity assay

#### Collection of bacterial supernatants

Overnight cultures of the selected *B. cereus* group isolates grown in TSB were transferred into sterile Erlenmeyer flasks with 30 ml TSB until the OD values at 600 nm reached 0.13–0.14. The flasks were incubated under continuous shaking conditions (150 rpm) at 30°C for 6–8 h until the early-stationary growth phase reached an OD_600_ of 1.0 (equivalent to 10^8^ CFU/ml). The cultures were centrifuged at 10,000 *×g* for 5 min, then the supernatants were collected and filter sterilized using 0.2 μm membrane filters connected to a syringe.Sterile supernatant aliquots of 1 ml were stored at −75°C, and used for further analyses within 4 weeks (Shi et al., 2016; Johler et al., 2018; Huang et al., 2019). Bc 836 was used as the positive control in the analyses (Table 1). Three batches of supernatants from each isolate were collected independently on three different days.

#### Hemolysin BL enterotoxin detection

The BCET-RPLA toxin detection kit (Oxoid) was used to detect the L2 fragment of Hbl (a subunit of enterotoxin Hbl). The reversed passive latex agglutination (RPLA) technique enables soluble antigens (bacterial toxins in this case) to be detected in an agglutination assay. Collected supernatants from selected isolates were adjusted to room temperature and used for the detection following the protocol provided by the manufacturer.

#### MTT-based cytotoxicity assay using intestinal Caco-2 cell line

To assess the cytotoxicity of selected isolates, Caco-2 cells with a density of 2 × 10^4^ cells/well were seeded in 96-well microplates containing DMEM (high glucose) with 10% FBS and 1% NEAA and incubated at 37°C with 10% CO_2_ for 18–24 h. After incubation, the medium in each well was carefully removed and treated with 200 μl DMEM containing 1% v/v of bacterial supernatants, which were thawed and pre-warmed to 37°C. The wells only treated with DMEM were used as the untreated controls. Four wells per treatment were used in each independent experiment. After incubation at 37°C with 10% CO_2_ for 24 h, 100 μl medium from each well was carefully removed and 20 μl 3-[4,5-dimethylthiazol-2-yl]-2,5-diphenyl tetrazolium bromide ([MTT]; Invitrogen, United States) reagent (5 mg/ml; filter sterilized) was added. The microplates were incubated in the dark at 37°C with 10% CO_2_ for 2 h, and then the liquid in each well was carefully removed using a multichannel pipet. Subsequently, 200 μl Dimethyl sulfoxide (DMSO; Sigma-Aldrich, United States) was added to each well and suspended using a multichannel pipet until complete solubilization of the blue formazan crystals. The absorbance was measured in a SpectraMax microplate reader (Molecular Devices, United States) at 570 nm. The experiments were repeated with three different batches of supernatants per isolate giving three independent biological replicates. The corrected absorbances (subtracted from the background signal of the blank group) were used in the calculation of the cell viability determined by calculating a ratio of each treatment to the untreated control. Cell viability was expressed as the average percentage of the three biological replicates of each isolate. Isolates were classified as having low (66.7–100% cell viability), medium (33.3–66.7% cell viability) or high (0–33.3% cell viability) cytotoxicity. The classification of the isolates based on reciprocal titers was also normalized using the cytotoxic reference strain ATCC 14579 (type strain, Bc 836 in our study; Kilcullen et al., 2016).

### Statistical analyses

All statistical analyses were performed in SPSS Statistics 27 software (IBM SPSS Statistics, United States). For quantitative variables, i.e., bacterial counts, OD values and ratio numbers of cytotoxicity, an independent t-test or one-way analysis of variance (ANOVA) was performed. The bacterial counts and ratio numbers were transformed into log values for statistical analyses. The normality and equality of variances were checked by Shapiro–Wilk test and Levene’s test, respectively. Differences between two groups of independent samples were analyzed by an independent two-sided *t*-test (*α* = 0.05), and differences among three or more groups of independent samples were analyzed by one-way ANOVA (*α* = 0.05) following an applicable *post-hoc* test for multiple comparisons. If data (or transformed data) followed a normal distribution with homogeneous variances, then Bonferroni *post-hoc* test was performed, resulting in adjusted *p* values. In order to check the association between pre-harvest Bt treatment and post-harvest Bt prevalence (%), Fisher’s Exact tests (two-sided) were performed. Differences were considered to be significant at levels of *p* < 0.05.

## Results

### Occurrence and residues of presumptive *Bacillus cereus* and Bt from fresh to frozen spinach

Presumptive *B. cereus* was found in all Bt-treated fresh and frozen samples with counts in a range of 1.95 × 10^4^–1.75 × 10^5^ CFU/g and 2.5 × 10^2^–1.15 × 10^3^ CFU/g, respectively (Table 4; Supplementary Table 1). Bt was found in all these pre-harvest Bt-treated samples, all 17 (100%) isolates from fresh spinach and 47 out of 49 (96%) isolates from frozen spinach were identified as presumptive Bt by the presence of parasporal crystals (Table 4). It is worth noting that the residues of presumptive *B. cereus* from one fresh spinach sample exceeded the threshold of 1 × 10^5^ CFU/g. This sample was treated with Bt biopesticides (XenTari^®^) in the field 2 days before harvest. The three isolates collected from this sample were identified as presumptive Bt (Supplementary Table 1). In contrast, 35 samples that had not been treated with Bt biopesticides in the field were found to possess presumptive *B. cereus* with the prevalence of 50, 67 and 33% in fresh (*n* = 14), fresh washed (*n* = 9) and frozen (*n* = 12) samples, respectively. The counts were 2–3 log lower in the non-Bt-treated fresh spinach samples compared to the fresh spinach samples, which were sprayed with Bt biopesticides (XenTari^®^) 2 days before harvest in the field (Table 4; Supplementary Table 1). Only 100 CFU/g presumptive *B. cereus* were detected in all four positive frozen samples that had never been sprayed with Bt biopesticides in the field. This count was at the limit of detection corresponding to 1 colony on the MYP-spread plate that was inoculated with 100 μl of the initial 10-fold diluted suspension of spinach. None of the isolates from these non-Bt-treated fresh spinach samples were found with parasporal crystals by microscopy. However, 87% (20/23) and 75% (3/4) of the isolates from these non-Bt-treated fresh washed and frozen samples were identified as presumptive Bt by the production of parasporal crystals.

**Table 4:** Prevalence of presumptive *B. cereus* and Bt in spinach.

Presumptive *B. cereus*	Fresh spinach from field (*n* = 18)		Fresh washed spinach (*n* = 11)		Frozen spinach (*n* = 20)	
	Treated with Bt (*n* = 4)	Non-treated with Bt (*n* = 14)	Treated with Bt (*n* = 2)	Non-treated with Bt (*n* = 9)	Treated with Bt (*n* = 8)	Non-treated with Bt (*n* = 12)
Prevalence	4/100%	7/50%	0/0%*	6/67%	8/100%	4/33%
Minimum counts in positive samples (CFU/g)	1.95 × 10^4^	100	<100*	100	250	100
Maximum counts in positive samples (CFU/g)	1.75 × 10^5^	850	<100*	800	1,150	100
Positive samples containing presumptive Bt	4/100%	0/0%	0/0%	6/100%	8/100%	3/75%
Number of isolates	17	20	0	23	49	4
Isolates identified as presumptive Bt	17/100%	0/0%	0	20/87%	47/96%	3/75%

* Unreliable results due to too many yellow colonies on MYP agar.

Overall, higher Bt residual numbers were observed from the fresh (independent *t*-test; *p* < 0.001) and frozen (independent *t*-test; *p* < 0.001) spinach samples that were treated with Bt biopesticides, than the samples which were never treated with Bt. In addition, there was a statistically significant association between Bt biopesticide treatment and Bt positive sample frequency in fresh (Fisher’s Exact test; *p* = 0.003) and frozen (Fisher’s Exact test; *p* = 0.001) spinach. Thus, the pre-harvest treatment of Bt biopesticides in the field can result in a higher prevalence (%) of Bt in both fresh and frozen spinach products.

### Identification of Bt biopesticides and other *Bacillus cereus* group isolates by WGS and qPCR assays

The 34 selected isolates (described in see section “*Bacillus cereus* group isolate selection and DNA preparation”) and four isolates from the Bt biopesticides (XenTari^®^, Turex^®^ WG, Dipel^®^, and Delfin^®^ WG) were subjected to WGS. The overall genomic characteristics and Benchmarking Universal Single-Copy Ortholog (BUSCO) assessments of the assemblies from these isolates are shown in Supplementary Table 4; Supplementary Figure 1. Isolate B22 was discarded for further analyses as it was a mixture of *B. cereus* group and *Lactococcus* spp. Because high fragmented BUSCOs (14.6%) were identified in the assembly of turex (strain collection number GC-91), the assembly of turex was omitted. Instead, the high-quality genome of strain GC-91 was downloaded from NCBI database (accession number: GCA_020809205.1), and was included in downstream analyses. Because this strain was isolated from the commercial product Agree^®^ 50 (Biggel et al., 2022a), thus it was called as agree 50 in our study to easily link the isolate to the commercial product.

**Figure 1:**
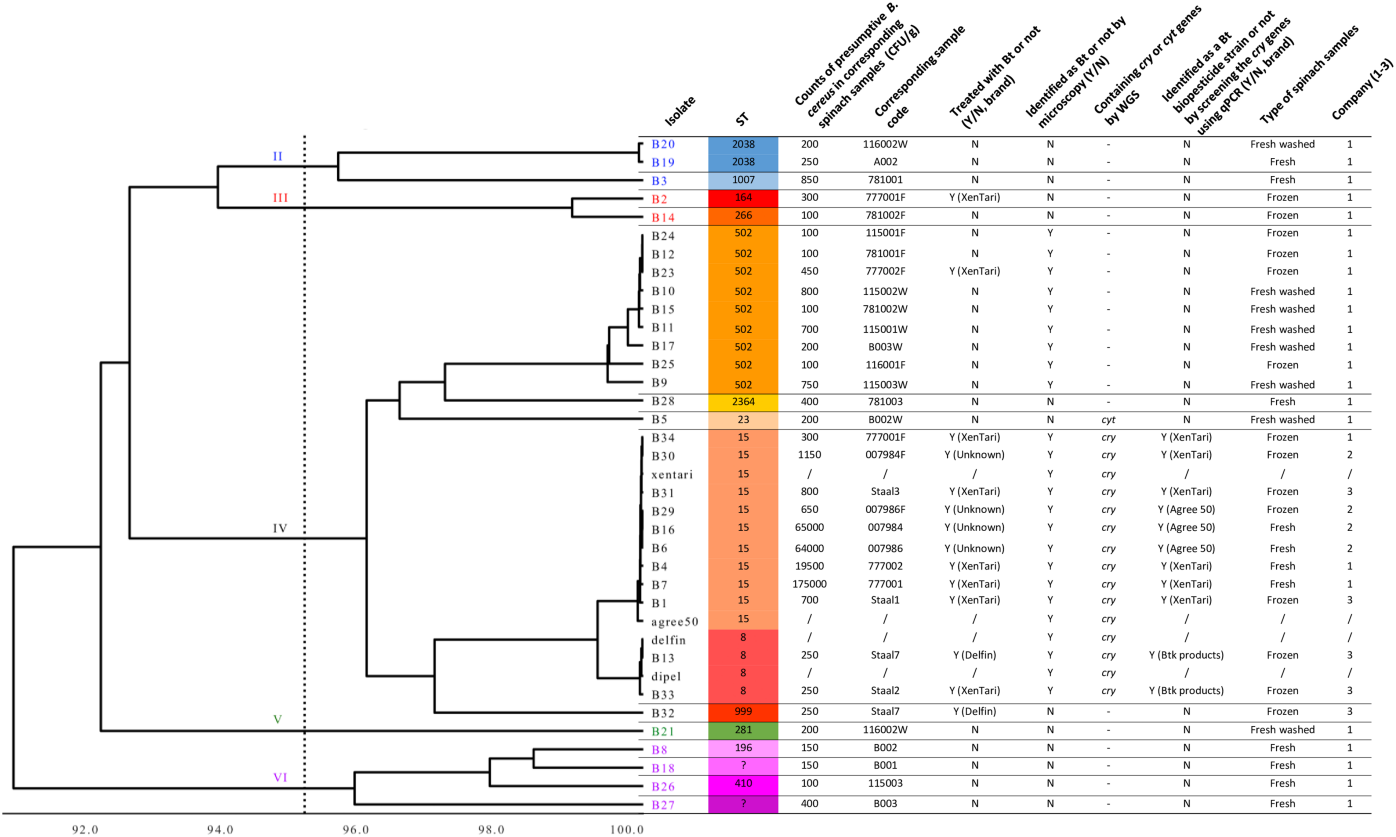
MASH-ANI tree of 33 spinach isolates and 4 Bt biopesticide isolates. The scale of the tree indicates the Average Nucleotide Identity (ANI) values, and the vertical dashed line shows the cut-off value (95) used traditionally to differentiate among bacterial species. The high-quality genome of agree 50 was downloaded from NCBI database (Biggel et al., 2022a). The Roman numerals labeled on the tree indicate different *panC* groups. “Y” represents “Yes” and “N” represents “No.” “/” represents “not applicable.” “–“ represents “not detected.”

Whole-genome comparisons using ANI were performed in dRep and shown as a distance tree in Figure 1. This MASH-ANI tree indicated the division of these isolates into five groups corresponding to II-VI *panC* clades (Guinebretière et al., 2008) identified by BTyper 3. WGS-based MLST identified 15 STs of all tested isolates. Three STs containing multiple isolates were identified: nine spinach isolates were assigned to ST 502, nine spinach isolates and two Bt biopesticide isolates (xentari, agree 50) were assigned to ST 15, and two spinach isolates and another two Bt biopesticide isolates (dipel, delfin) were assigned to ST 8 (Figure 1). All 15 isolates assigned to ST 15 and ST 8 were positive for *cry* genes reported by both BTyper 3 (Supplementary Table 5) and PROKKA (Table 5), and B5 isolate assigned to ST 23 was only positive for *cyt* genes reported by both two methods. Therefore, these 16 isolates including 12 spinach isolates and four Bt biopesticide isolates were identified as Bt (Figure 1). In addition, 11 out of 33 spinach isolates were found positive for *cry* genes screened by qPCR test and then identified as the commercially available Bt biopesticide strains (Figure 1; Supplementary Table 6).

**Table 5:** Gene content of the *cry* and *cyt* genes identified by WGS annotation in the 16 isolates.

	ST 23	ST15										ST 8				
Isolate	B5	B34	B30	xentari	B31	B29	B16	B6	B4	B7	B1	agree 50	B13	delfin	dipel	B33
*cry1Aa*																
*cry1Ab*																
*cry1Ab_1*																
*cry1Ab_2*																
*cry1Ac*																
*cry1Ca*																
*cry1Da*																
*cry1Ia*																
*cry2Aa*																
*cry2Ab*																
*cry9Ea*																
*cyt2Aa1_1*																
*cyt2Aa1_2*																

Blue shading indicates that a gene was detected from automatic annotations of the assemblies performed using PROKKA under default settings.

Bt genes (*cry*, *cyt*, or *vip* genes) were not detected by WGS and qPCR in the isolates identified as non-Bt strains by phase-contrast microscopy, except for B5 in which *cyt* genes were detected by WGS (Figure 1). Interestingly, isolates assigned to ST 502 in *panC* clade IV were observed with parasporal crystals, however, *cry* or *cyt* genes were neither detected by WGS nor by the qPCR assay. The Bt isolates (*n* = 11) that harbored *cry* genes from Bt-treated spinach samples were all assigned to the same sequence types (ST15 and ST8) with the Bt biopesticide isolates. In these 11 Bt isolates, six (B34, B31, B4, B7, B1, and B13) clustered together (>99.9% ANI) with the same isolate from the Bt biopesticide (XenTari^®^ or Delfin^®^) used in the field (Figure 1). Identification results of these six spinach isolates using the qPCR assay were also in line with the information provided by the food companies (Supplementary Table 6; Figure 1).

However, isolate B33 from XenTari^®^ treated spinach frozen samples clustered together (>99.9% ANI) with *B. thuringiensis* subspecies *kurstaki* (Btk) strain delfin and dipel in ST 8, instead of xentari in ST 15 (Figure 1). The qPCR assay also identified B33 as a Btk isolate, which was consistent with the results of WGS. It is also worth noting that the qPCR identification results of B16 and B30 were different (Figure 1). However, WGS results indicated B16 and B30 were both closer to xentari (genome-wise, based on ANI) rather than agree 50 (Figure 1). In addition, the corresponding fresh and frozen samples of B16 and B30 were from the same batch spinaches sprayed once with Bt biopesticides in the same field provided by Company 2. While no information about the brand of used Bt products was provided by Company 2 to clarify the conclusion, and xentari and agree 50 were also genomically similar to each other sharing >99.9% ANI. Similarly, B6 and B29 from the same batch of spinach in the field showed the closest similarity with xentari (based on ANI), but were both identified as GC-91 by qPCR assay (Figure 1; Supplementary Table 6).

SNP analysis showed that the wgSNP distances between nine spinach isolates in ST 15 and xentari ranged from 0 to 4 (mean of 1.9), and between two spinach isolates in ST 8 and delfin was 8 to 12 (mean of 10) as shown in Table 6; Supplementary Tables 7, 8. Combining all results mentioned above (sampling information, ANI and SNP analysis), xentari was considered as the identical Bt strain for the spinach isolates from ST 15, and delfin was considered as the identical Bt strain for spinach isolates from ST 8.

**Table 6:** Whole-genome SNP distances between spinach Bt isolates and their closest Bt biopesticide strain using CFSAN pipeline.

Isolate	ST	wgSNP distance to the closest biopesticide isolate^*^
xentari	15	–
B1	15	2
B4	15	0
B6	15	3
B7	15	2
B16	15	2
B29	15	1
B30	15	2
B31	15	4
B34	15	1
delfin	8	–
B13	8	8
B33	8	12

* indicates the pairwise wgSNP distance between spinach Bt isolates assigned to ST 15 and the closest biopesticide strain xentari from the same ST; or the pairwise wgSNP distance between spinach Bt isolates assigned to ST 8 and the closest biopesticide strain delfin from the same ST.

### Lowest growth temperature of *Bacillus cereus* group isolates from spinach

Six isolates (B20, B3, B8, B18, B26, and B27) grew at a temperature < 8°C, while B8, B18, B26 grew at 4°C. These psychrotolerant isolates were assigned to *panC* group II and VI. Isolates allocated to ST 15 and ST8, including the four Bt biopesticide isolates, cannot grow at <8°C. The lowest growth temperature of all the spinach isolates included in this study assigned to *panC* group III, IV and V is 10°C (Table 7).

**Table 7:** Characterization of 28 selected *B. cereus* group isolates from Bt biopesticides and spinach samples obtained in this study.

Isolate	*panC* group	ST	Corresponding counts of *B. cereus s.l.* in spinach samples (CFU/g)	Type of spinach samples	Toxin profile^a^^,^^b^	Lowest growth temperature (°C)	Biofilm formation capacity^c^		Cytotoxicity^d^		
							OD (mean)	Classification	Reciprocal titer	Normalized reciprocal titer	Classification
B20^*^	II	2038	200	Fresh washed	C	7	3.18	S	1.00	0.50	Low
B3	II	1,007	850	Fresh	F	7	0.25	W	1.24	0.62	Low
B2	III	164	300	Frozen	F	10	0.78	S	3.07	1.55	High
B14	III	266	100	Frozen	F	10	0.45	M	251.00	126.34	High
B12	IV	502	100	Frozen	A	10	0.14	N	1.07	0.54	Low
B23	IV	502	450	Frozen	A	10	0.15	N	1.01	0.51	Low
B15	IV	502	100	Fresh washed	A	10	0.14	N	0.98	0.49	Low
B11	IV	502	700	Fresh washed	A	10	0.14	N	1.01	0.51	Low
B25	IV	502	100	Frozen	A	10	0.86	S	1.01	0.51	Low
B9	IV	502	750	Fresh washed	A	10	1.73	S	0.96	0.48	Low
B28	IV	2,364	400	Fresh	A	10	2.58	S	1.38	0.69	Low
B5	IV	23	200	Fresh washed	C	10	3.68	S	341.67	171.98	High
xentari	IV	15	/	/	A	10	0.50	M	1.00	0.50	Low
B31	IV	15	800	Frozen	A	10	0.55	M	0.99	0.50	Low
B29	IV	15	650	Frozen	A	10	0.47	M	0.98	0.49	Low
B1	IV	15	700	Frozen	A	10	0.46	M	0.97	0.49	Low
turex	IV	15	/	/	A	10	0.68	S	1.06	0.53	Low
B13	IV	8	250	Frozen	A	10	0.28	W	1.74	0.88	Mid
delfin	IV	8	/	/	A	10	0.33	M	1.42	0.71	Low
dipel	IV	8	/	/	A	10	0.21	W	1.70	0.86	Mid
B33	IV	8	250	Frozen	A	10	0.29	W	1.63	0.82	Mid
B32	IV	999	250	Frozen	A	10	3.81	S	4.07	2.05	High
B21	V	28	200	Fresh washed	C	10	0.40	M	1.20	0.60	Low
B8	VI	196	150	Fresh	C	4	3.27	S	1.06	0.53	Low
B18	VI	?	150	Fresh	C	4	0.35	M	1.00	0.51	Low
B26	VI	410	100	Fresh	C	4	3.00	S	1.04	0.52	Low
B27	VI	?	400	Fresh	C	7	2.41	S	1.09	0.55	Low

a Toxin profiles A-G represent the presence of the following toxin genes patterns: A, *nhe, hbl, cytK*; B, *nhe, cytK, ces*; C, *nhe, hbl*; D, *nhe, cytK*; E, *nhe, ces*; F, *nhe*; G, *cytK* (Ehling-Schulz et al., 2006).

b Only *cytK-2* was detected in the *cytK* positive isolates.

c Categorization of isolates based on biofilm-forming capacity: N, non-biofilm former (OD ≤ 0.15); W, weak biofilm former (0.15 < OD ≤ 0.3); M, moderate biofilm former (0.3 < OD ≤ 0.6); S, strong biofilm former (OD > 0.6).

d Cytotoxicity classification of the supernatants from the isolates was normalized using the absolute value of the reference strain ATCC 14579. Isolates were classified as low (<0.8), mid (0.8–1.5) or high (>1.5) level of cytotoxicity with the adaptation of Johler et al. (2018).

* A truncated *hb*l operon was detected by Btyper 2, but this isolate was able to produce Hbl L2 (positive in BCET-RPLAs), thus it was allocated to toxin profile C and A.

“/” represents “not applicable.”

### Biofilm formation capacity of *Bacillus cereus* group isolates from spinach

**Figure 2:**
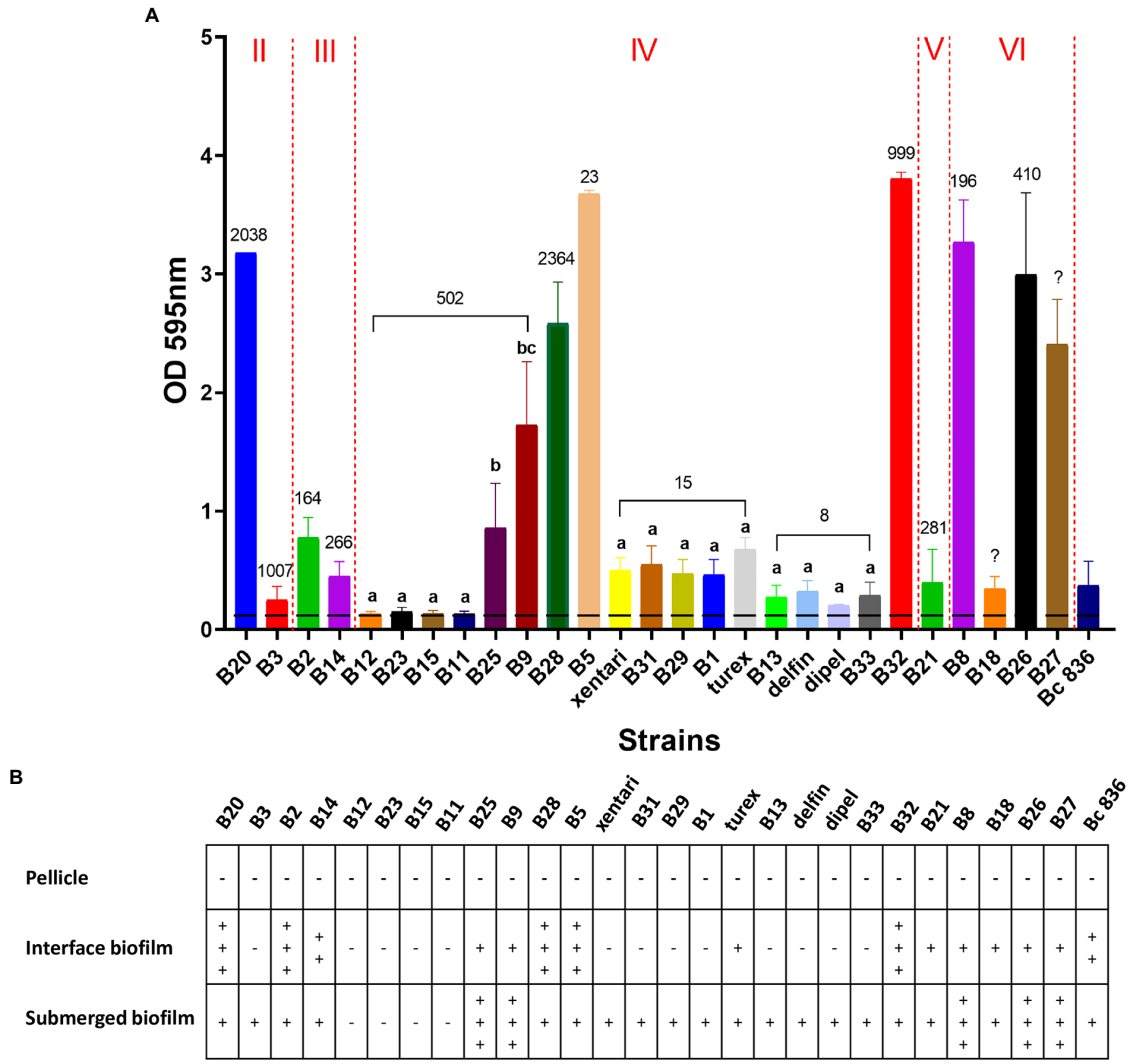
Biofilm forming ability of 23 spinach isolates and 4 Bt biopesticide isolates in TSB on polystyrene for 24 h at 30°C. Bc 836 (ATCC 14579) worked as the positive control. **(A)** OD values at 595 nm obtained by CV assays. Data are presented as mean ± standard deviation (SD). The horizontal dashed line indicates the background ODs from the negative control. The Roman numerals labeled on the top indicate different *panC* groups, the numbers labeled on the bars represent the STs. Bars sharing common letters are not significantly different from each other (*p* ≥ 0.05). **(B)** Different types of biofilm formed by different strains. “–” means negative, “+” to “+++” shows “weak” to “strong” visible biofilm observed.

In total, 23 out of 27 isolates were able to form biofilm, only B12, B23, B15, and B11 from ST 502 were non-biofilm formers (Figure 2A; Table 7). No significant differences (Bonferroni; *p* > 0.05) of OD values were observed among isolates in ST 15 and ST 8 containing Bt biopesticide strains (Figure 2A). Although turex was characterized as the only strong biofilm former in ST 15 and delfin was the only moderate biofilm former (Table 7). The biofilm-forming capacity of B25 and B9 were both significantly stronger (Bonferroni; p < 0.001) than the other four isolates from ST 502 (Figure 2A). No significant differences (Bonferroni; p > 0.05) were detected either between B25 and B9 or among B12, B23, B15, and B11 (Figure 2A). Different biofilm types formed by the isolates are also elaborated in our study (Figure 2B). After the incubation in TSB for 24 h at 30°C, no visible floating pellicles were observed in all tested isolates. Strong air-liquid biofilms were formed by B20, B2, B28, B5, and B32, while strong submerged biofilms were formed by B25, B9, B8, B26, and B27. It is also worth noting that turex formed the air-liquid interface biofilm on the wall of wells, while xentari and the other isolates in ST 15 did not form the air-liquid interface biofilm after incubation at 30°C for 24 h. This indicates that the spinach isolates are different strains from turex and most likely closely related to xentari, which is consistent with the WGS results mentioned in see section “Identification of Bt biopesticides and other *B. cereus* group isolates by WGS and qPCR assays.” It can be concluded that all biofilm formers have the ability to form a submerged biofilm, not an air-liquid biofilm, and that strain-specific biofilm formation capacity has been observed.

### Toxin gene profiling and *in vitro* enterotoxin (Hbl) expression

**Figure 3:**
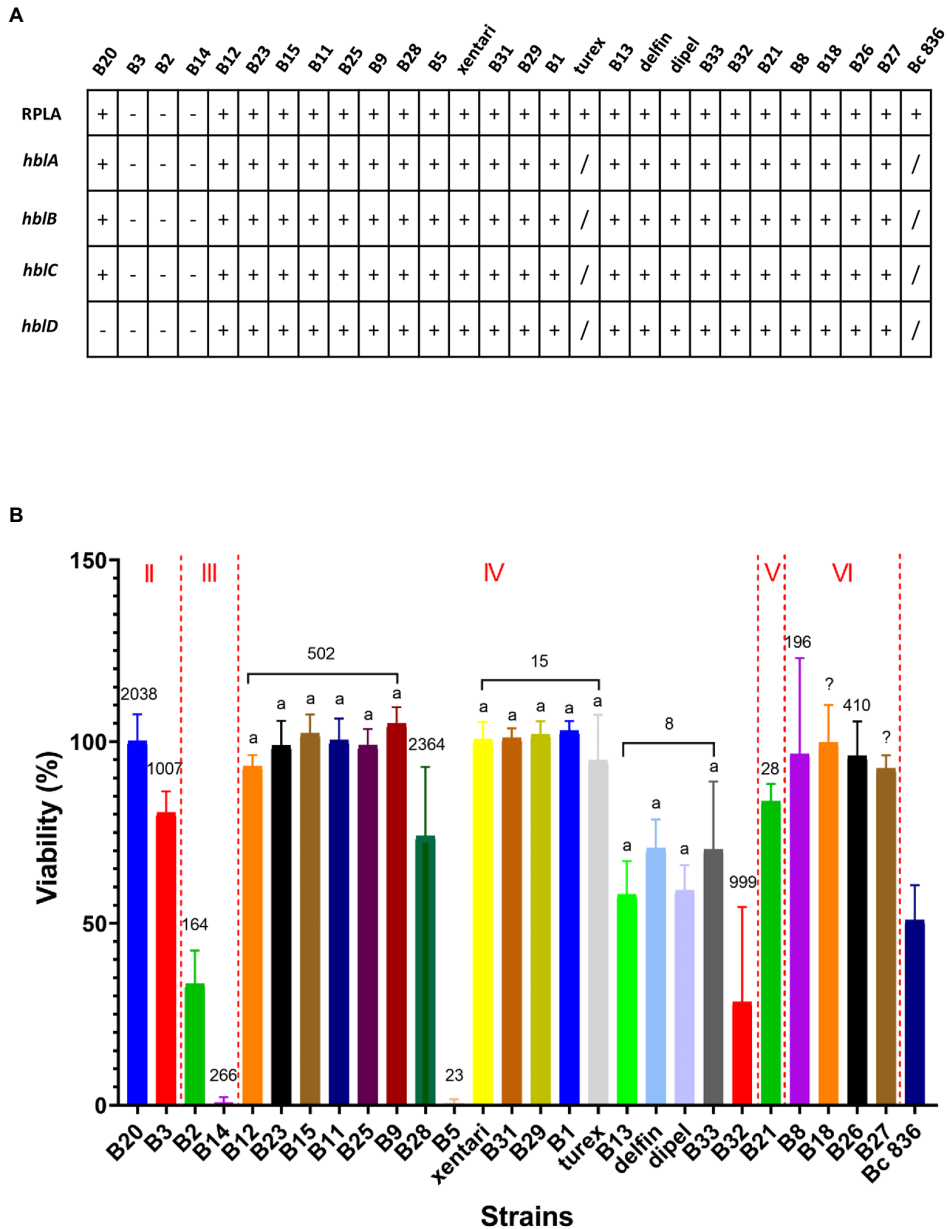
Hemolysin BL (Hbl) detection and cytotoxicity from bacterial culture supernatants. Bc 836 (ATCC 14579) worked as the positive control. **(A)** Hbl detection by BCET-RPLA assay and *hbl* genes detected *in silico* by WGS. “–” represents negative, “+” represents positive. **(B)** Cell viability (% relative to the untreated control) was used to determine the cytotoxicity on Caco-2 cells by MTT assays. Data are presented as mean ± SD of three biological replicates. The Roman numerals labeled on the top indicate different *panC* groups, the numbers labeled on the bars represent the STs. Bars sharing common letters are not significantly different from each other (*p* ≥ 0.05). “/” represents “not tested.”

No *ces* genes encoding for the synthesis of the emetic toxin cereulide and *cytK-1* genes encoding cytotoxin K-1 (CytK-1) were detected in any of the isolates, but all isolates across different clades harbor the majority of all virulence genes reported by BTyper 2.3.2 (Supplementary Table 9). For diarrheal toxin genes, all 37 tested isolates possessed at least one of the *nhe* genes (*nheA*, *nheB* or *nheC*) with high identity, and 34 out of 37 isolates were positive for at least three of the *hblABCD* genes except for B3 from *panC* group II, and B2 and B14 from *panC* group III. This was confirmed by the detection of Hbl in bacterial supernatants of *B. cereus* group isolates performed by the BCET-RPLA kit (Figure 3A). All isolates that harbored *hbl* genes expressed Hbl including the isolate (B20) which was found to possess a truncated *hbl* operon (Figure 3A). Only the isolates from *panC* group IV possessed the *cytK-2* gene except for B5, while the other isolates from *panC* group II, III, V and VI do not possess the *cytK-2* gene. Thus, only toxin profiles A, C and F were found in these isolates, and all Bt biopesticide isolates and the genomic similar isolates of the same STs were assigned to toxin profile A (*nhe, hbl, cytK*; Table 7).

### Cytotoxicity

Using 1% bacterial supernatants, 11 out of 27 tested isolates showed medium or high cytotoxic effects on Caco-2 cells, whereas the remaining 16 isolates showed limited effects on Caco-2 cells maintaining >90% viability (Figure 3B) and were accordingly classified as having low cytotoxicity (Table 7). No significant differences were observed among isolates within the same ST (ST 502, ST 15, and ST 8). Two highest cytotoxic isolates (B14 and B5) were identified in frozen and fresh-washed spinach samples from Company 1. Besides, B2 and B32 from frozen spinach from Company 1 and Company 3 were also characterized as high cytotoxic isolates. Although negative for both presence of *hbl* genes and the production of Hbl enterotoxins, cytotoxic effects on Caco-2 cells were observed by B2, B3, and B14 which might be contributed by the other enterotoxins (i.e., Nhe).

## Discussion

**WGS is still the best way to identify Bt biopesticide isolates at the strain level, but the developed qPCR assay by screening cry genes can be considered as a rapid routine test together with the identification of presumptive Bt by phase-contrast microscopy and supplementary sample information**. In this study, 11 out of 12 Bt isolates (tentatively identified by microscopy) from Bt-treated spinach samples showed high similarity with strains from Bt biopesticides, as evidenced by >99.9% ANI and ≤ 12 wgSNPs. In these 11 Bt isolates from spinach, the identification results of 8 isolates by qPCR assay were consistent with the WGS results. Six out of 11 Bt isolates originated from the applied Bt biocontrol products in the field (Figure 1). One isolate (B33) was identified as a Btk strain by WGS (>99.9% ANI; ST 8) and qPCR, while the used Bt biopesticide in the corresponding spinach sample was XenTari^®^ (active substance is xentari, a Bt *aizawai* strain). This isolate is likely to be originated from cross-contamination during post-harvest processing (sorting, washing, freezing, packing) in Company 2. Because it was from the frozen spinach sample processed in company 2 which also processed the Delfin^®^ treated spinach and delivered the samples to the lab in the same batch (Figure 1; Supplementary Table 1). Thus, delfin was considered as the identical Bt biopesticide strain of B33, and the wgSNP distance between B33 and delfin was 12. Until now, there is still no specific SNP cutoff value to define closely related isolates, whereas a distance of 0–10 core genome SNPs (cgSNPs) to biopesticide strains has been recently suggested as a reasonable range to assume a biopesticidal source of food isolates using CFSAN (Biggel et al., 2022b). However, our results showing 0–12 wgSNP distances might be slightly higher than the cgSNPs threshold (10 cgSNPs). This higher wgSNP difference was also reported by one recent study: Biggel et al. (2022a) revealed that 20 obtained Bt isolates from food and outbreaks genomically matched (≤6 wgSNPs, ≤2 cgSNPs; CFSAN) six different biopesticide strains. Thus, a distance of 12 wgSNPs could be considered as the cutoff value to define identical Bt biopesticide isolates.

For four Bt isolates from Bt-treated spinach samples provided by Company 2, no detailed information on the brand of used Bt biopesticide product was provided, SNP analysis identified xentari as the identical (≤3 wgSNPs) Bt biopesticide strain. One isolate (B30) showed consistent results from WGS and qPCR assay, whereas for the other three isolates (B29, B16, B6), qPCR identifications were different from the WGS results. By checking the *cry* gene contents identified by WGS annotation (Table 5), *cry1Ab* is also absent in the genome of B29, B16, and B6 but present in the genome of B30. While the qPCR assay used the screening of *cry1AaAb* as one target gene to differentiate the Bt isolates from Xentari^®^ and Agree^®^ 50. This explained the reason why the qPCR assay identified B29, B16 and B6 as the same isolate from Agree^®^ 50. The absence of the *cry1Ab* gene in these three isolates might be caused by the loss of a plasmid, as *cry* genes are located on plasmids, and the loss of plasmids with *cry* genes has been described to happen (Gonzalez et al., 1982; Höfte and Whiteley, 1989). Therefore, the qPCR assay can provide an indication that strains may be identical to the Bt biopesticide strains, while WGS is more adequate for the identification. Our results also showed that false-positive identification of Bt by phase-contrast microscopy might occur (i.e., the isolates from ST 502), which is consistent with other studies (EFSA, 2016; Nair et al., 2018) where some strains observed to possess parasporal crystals showed negative detection of *cry* and *cyt* genes. However, it cannot be ruled out that WGS and qPCR missed potential Bt toxin-encoding genes, which contributed to the production of crystals.

It is shown in this study that the developed qPCR assay by screening the *cry* genes can be used not only to identify the Bt isolate but additionally useful to track the use of authorized Bt BCAs on crops in the EU. Although it cannot be excluded that among the wide natural variety of Bt strains there might also be a strain that has the same *cry* gene profile as used for the identification of Bt biopesticide strains. Overall, the combination of this qPCR assay with microscopical identification and sampling information (e.g., organic production or not, pre-harvest treated with Bt biopesticides or not) can target the Bt strain derived from post-harvest food products to the pre-harvest use of Bt biopesticides. This can be of interest in food traceability studies or as part of routine food diagnostics. On the other hand, and particularly for foodborne outbreaks and clinical surveillance, generating high-throughput sequencing data and compare them with the complete (or near-complete) genomes is the best way to identify Bt at strain level.

**The use of Bt biopesticides on spinach plants in the field led to higher Bt residues (occurrence and numbers) in fresh spinach and even in frozen spinach products**. Recent studies also indicated a biopesticidal origin of Bt in fresh produce by WGS (Frentzel et al., 2020; Bonis et al., 2021; Biggel et al., 2022a), but without having any information on the use of Bt biopesticides in the plant. Frentzel et al. (2020) highlighted that Bt strains were the dominant isolates (>90%) recovered from the presumptive *B. cereus* positive bell pepper and tomato samples in Germany with a prevalence of 41 and 28%, respectively. SNP analyses indicated the genetic close relationship of 7 isolates with dipel (≤4 cgSNPs) and 18 isolates with xentari (≤2 cgSNPs) in total 42 tested isolates. However, only 2 out of 7 Bt biopesticides strains of the approved active substances used against insects on the crops in the EU (EU Pesticides Database, 2022) were included in that study. Thus, the identification of the potential use of the commercial biopesticides containing the other five Bt biopesticide strains on the tomato or bell pepper plants was overlooked. Bonis et al. (2021) reported that 49 foodborne outbreaks (FBOs) in France (19.6%, 2007–2017) were likely to be associated with Bt. In 154 FBO-associated Bt isolates, 137 isolates were clustered together with all Bt biopesticide isolates from *panC* group IV compared by SNP-based core-genome phylogenetic analysis. In these 49 FBOs, the reported Bt levels ranged from 1 × 10^2^ to more than 1 × 10^7^ CFU/g (median of 9 × 10^2^), and >50% of Bt isolates were from dishes containing raw vegetables. However, more evidence should be provided to support Bt was the only causative agent with low residual numbers (i.e., <1,000 CFU/g). Biggel et al. (2022a) also investigated the prevalence of Bt in fresh produce at the retail level in Switzerland; 14 (with 3 leafy vegetable samples) out of 100 food samples were found to contain Bt (all < 10^5^ CFU/g). Further, they found that 49 (63%) of the collected isolates were identified as Bt by phase-contrast microscopy, and 20 food and outbreak-associated Bt isolates were identical with six Bt biopesticide strains using SNP analysis However, limited studies on the prevalence of Bt specifically in leafy vegetables have been reported. Kim et al. (2017) showed that 25 (96.2%) of 26 organic leafy vegetable samples in South Korea were detected with presumptive *B. cereus* with a mean of 3.51 log CFU/g, of which 23 (88.5%) samples were found with Bt by the detection of crystal proteins and *cry* genes. As BCAs play a critical role in organic farming as alternatives, where synthetic fertilizers and pesticides are not permitted. Thus, this high reported occurrence of Bt in organic leafy vegetables by Kim et al. (2017) might also originate from the use of Bt biopesticides, whereas additional information about Bt treatment or advanced molecular identification is needed to confirm this hypothesis.

**Psychrotolerant properties of the B. cereus group isolates were specifically detected in panC groups II and VI**. In this study, all isolates assigned to *panC* group II and VI have the ability to grow at 7°C and even at 4°C, which confirms previous studies (Guinebretière et al., 2008; Beno et al., 2019). The closest strains identified based on ANI vaules were from either the GTDB species *B. wiedmannii* or *Bacillus weihenstephanensis* (data not shown), both species have been reported as psychrotolerant (Lechner et al., 1998; Miller et al., 2016). These recognized psychrotolerant *B. cereus* group isolates have the potential to cause food safety or quality problems during refrigerated storage and during the distribution process of food products (Park et al., 2020).

**Cytotoxicity seems to be associated with MLST sequence types**. Using cell-free supernatant to assess the cytotoxicity is a fast and simple test to evaluate the pathogenic potential of *B. cereus* in humans (Glasset et al., 2021). Different isolates from the same ST (i.e., ST 502, ST 15, or ST 8) in our study were characterized by similar cytotoxic activities, although isolates from the same clone which can be identified as the same strain were also found in each ST. Thus, a greater number of different strains should be studied in each ST in the future to confirm this hypothesis that cytotoxicity might be associated with MLST sequence types. However, the choices of different cell lines and supernatant dilutions may impact the outcome and thus need further elaboration in future studies. Cytotoxicity of Bt enterotoxins in supernatants to Vero or Caco-2 cells was also reported: low to high cytotoxic effects were determined but no correlation between cytotoxicity and ST was ever checked (Gaviria Rivera et al., 2000; Johler et al., 2018; Schwenk et al., 2020).

**Strain-specific biofilm formation capacity was observed**. Since *B. cereus* group strains are widespread in the environment and are often found on food contact surfaces, the determination of biofilm-forming capacity of these strains is crucial to indicate their potential to cause persistent contaminations (Majed et al., 2016). *B. cereus* group strains have been reported with the ability to form three different types of biofilm including floating pellicles, air-liquid and submerged biofilm (Wijman et al., 2007). Most tested isolates were able to form biofilm, but unlike cytotoxicity, more strain-specific biofilm-forming abilities and types were observed (i.e., six isolates in ST 502) and as such biofilm formation can be used as an indicator for identifying differences among strains within the same ST. Four tested non-biofilm formers (B12, B23, B15, and B11) from ST 502 together with B24 and B10 from the same clone (Figure 1) were all recovered from fresh washed or frozen spinach samples from Company 1, but not from fresh spinach samples from the field. This might indicate that the origin of these frequently detected isolates is situated within the processing company 1 and cross-contaminated, e.g., *via* water in the washing tank (Banach et al., 2015) or from the food contact surfaces in this company. It is the first time that four Bt biopesticide strains are reported as biofilm formers, which indicates their potential to cause persistent contaminations in the environment of food processing companies.

**To evaluate the likelihood of the enteropathogenic B. cereus group strain causing disease upon consumption of the final food product, the residual number exposed to consumers is still an important index that should be considered. Comprehensive characterization of the isolates and the evaluation of their growth potential in the food supply chain should be established**. As the virulence genes are widely distributed across the clades, and no clear correlations between the toxin gene profiles and cytotoxicity exist (Jeßberger et al., 2015; Miller et al., 2018), more phenotypic traits should be evaluated to identify and characterize the hazard of the tested microorganism (i.e., growth ability at refrigerator temperatures, biofilm-forming capacity, survival during the passage of the stomach, ability to germinate and grow in the small intestine, enterotoxin production under gastrointestinal environment and cytotoxicity, etc.; Jessberger et al., 2020). Even though Bt is identified as a foodborne hazard, its manifestation generally results in rather mild self-limiting symptoms (FAO, 2012), and non-hospitalization of patients of this type of foodborne diarrheal toxico-infection. If humans are exposed to numbers of this enteropathogenic isolate lower than 10^5^ CFU/g, the likelihood to cause severe disease will be usually identified as low (EFSA, 2016; De Bock et al., 2021). It has been taken into account that the presence of enterotoxin indicates pathogenic potential but the toxin gene expression and enterotoxin production in consumer’s intestines depends upon many parameters (Ceuppens et al., 2011). In the present study, one non-biopesticidal origin Bt isolate (B5; *panC* group IV, ST 23) has quite high cytotoxicity to Caco-2 cells and thus indicates the functionality of enterotoxin genes. This Bt isolate can form strong biofilms and can thus be likely to be persistent in the food processing production environment, but it has no ability to grow at refrigeration temperatures (4°C or 7°C) common in use in the food supply chain. Thus, B5 isolate is not expected to germinate and multiply in cold conditions during standard post-harvest storage if the isolates are present on the end product (fresh or frozen spinach). The corresponding residual numbers in the fresh-washed spinach sample of B5 were determined to be 200 CFU/g (much lower than 10^5^ CFU/g). Therefore, the likelihood to cause toxico-infection for consumer upon ingestion of this fresh-washed spinach sample is low, and this sample would as such be regarded as safe for consumption by food safety authorities. This is an example of how food safety risk analysis combines the input of various sources of information related to the hazard but also related to the exposure to the hazard upon consumption for the risk assessment (Stewart et al., 2016).

It is of interest to note the following observations from the present study related to the Bt biopesticide strains: ST 8 isolates (with delfin and dipel) cannot grow at standard foreseen refrigerator temperatures, have low to moderate biofilm-forming ability and cytotoxic activity; Isolates in ST 15 (with xentari) cannot grow at refrigerator temperatures, have moderate biofilm-forming ability and low cytotoxic activity. However, elevated numbers of Bt were noted in fresh spinach samples that were pre-harvest treated with Bt biopesticide. Most samples showed numbers below 10^5^ CFU/g and in a case-by-case risk analysis might be considered as safe (enough) by food safety authorities. However, residual numbers of the Bt biopesticide strains (Bt isolates from ST 15, identical with xentari) of one fresh spinach sample (sample 777,001) were higher than the present commonly assumed safety limit of 10^5^ CFU/g. This sample would be not acceptable according to the food safety authorities in the current set framework of case-by-case risk analysis. As for this particular spinach batch information was available on the application of XenTari^®^ to the spinach plants in the field 2 days before the harvest, which can explain the reason for the high residual numbers of xentari in the fresh spinach samples exceeding the present commonly assumed safety limit of 10^5^ CFU/g. This suggests it is necessary to establish a longer pre-harvest interval to lower the likelihood of the occurrence of residual counts of Bt biopesticide strains exceeding 10^5^ CFU/g.

In conclusion, by combining information about the use of Bt biopesticides provided by the food processing companies, genomic similarity obtained from WGS-based data and *cry* genes profiles identified by the qPCR assay, six out of 11 Bt isolates originated from the applied Bt biocontrol products. It also reveals that the use of Bt biopesticides on spinach plants in the field could lead to higher residual numbers of Bt in spinach in the food supply chain (fresh or frozen). The risk and likelihood to cause food toxico-infection by the presence of elevated numbers of Bt strains that can *in vitro* express enterotoxin are still prone to debate. In a case-by-case risk analysis, the hazard needs appropriate identification and characterization, and the WGS, the qPCR assays and cytotoxicity testing can contribute to this. But also, the capacity to persist and grow in the food supply chain was exemplified in this study by assessing the growth potential at refrigeration temperatures and biofilm capacity. These two factors will impact whether Bt strains can be introduced from the field application into the production plant, and will also determine if these Bt strains can find an ecological niche in the food processing companies and the final product (in this case washed or frozen spinach). The actual number of Bt that consumers are exposed to is still an important index that should be considered in a microbiological food safety risk assessment. Although after pre-harvest Bt biopesticides treatment, exceeding the present commonly assumed safety limit for *B. cereus* group (including Bt) of 10^5^ CFU/g is rare in the present study, particularly for freshly washed or frozen spinach. It is therefore recommended to establish a pre-harvest interval for Bt biopesticide application in the field to lower the likelihood of noncompliance to this safety limit.

## Data availability statement

The datasets presented in this study can be found in online repositories. The names of the repository/repositories and accession number(s) can be found at: https://www.ncbi.nlm.nih.gov/genbank/, PRJNA858971.

## Author contributions

XZ conducted most of the experiments and data analysis and drafted the manuscript. AZ performed the WGS and bioinformatic analyses. MH performed the qPCR assays and related data analysis. MU, NH, LvO, and AR designed the study and revised the manuscript. All authors contributed to the article and approved the submitted version.

## Funding

This study was partially supported by the Ghent University, Aarhus University, and Wageningen University & Research. For WUR, design of the qPCR (TaqMan systems) was granted under TU18109. The project “distinction of *Bacillus thuringiensis* biocontrol from *Bacillus cereus sensu lato* strains in plant-derived food products” is a public-private collaboration granted by the topsector Horticulture and starting materials under TU18109.

## Conflict of interest

The authors declare that the research was conducted in the absence of any commercial or financial relationships that could be construed as a potential conflict of interest.

## Publisher’s note

All claims expressed in this article are solely those of the authors and do not necessarily represent those of their affiliated organizations, or those of the publisher, the editors and the reviewers. Any product that may be evaluated in this article, or claim that may be made by its manufacturer, is not guaranteed or endorsed by the publisher.

## Footnotes

^1^ https://github.com/FelixKrueger/TrimGalore

^2^ https://www.bioinformatics.babraham.ac.uk/projects/fastqc/
