# Supplementary material for: Identification and characterization of Bacillus thuringiensis and other Bacillus cereus group isolates from spinach by whole genome sequencing
Source: Front Microbiol. 2022 Nov 30;13:1030921. doi: 10.3389/fmicb.2022.1030921 (PMC9771606; doi:10.3389/fmicb.2022.1030921)
Supplement: Supplementary file 2 [file Table_2.DOCX]

**Table S2. Strains used in the development of the qPCR assay.**

| **Species** | **Subspecies** | **Strain** | **Origin** | **Provided by** |
| --- | --- | --- | --- | --- |
| *B. thuringiensis* | *kurstaki* | ABTS-351 | DiPel® | Valent BioSciences |
| *B. thuringiensis* | *kurstaki* | SA-11 | Delfin®WG | Certis |
| *B. thuringiensis* | *kurstaki* | SA-12 | Deliver WG® | Certis |
| *B. thuringiensis* | *kurstaki* | EG 2348 | Lepinox®Plus | Certis |
| *B. thuringiensis* | *kurstaki* | PB 54 | Belthirul® | Probelte |
| *B. thuringiensis* | *aizawai* | ABTS-1857 | Xentari® | Valent BioSciences |
| *B. thuringiensis* | *aizawai* | GC-91 | Agree® 50 WG | Certis |
| *B. thuringiensis* |  | ATCC 10792^T^ |  | - |
| *B. cereus sensu stricto* |  | ATCC 14579^T^ |  | - |
| *B. cereus sensu stricto* |  | NCTC 11143 |  | RIVM |
| *B. cereus sensu stricto* |  | F4810/72 |  | Aarhus University |
| *B. cereus sensu stricto* |  | AH187 |  | Aarhus University |
| *B. cereus sensu stricto* |  | NC7401/2455 |  | Aarhus University |
| *B. cereus sensu lato* |  | N5 |  | Aarhus University |
| *B. cereus sensu lato* |  | N6 |  | Aarhus University |
| *B. cereus sensu lato* |  | 2003/62 |  | Wageningen Plant Research |
| *B. cereus sensu lato* |  | 2003/63 |  | Wageningen Plant Research |
| *B. cereus sensu lato* |  | 2003/41 |  | Wageningen Plant Research |
| *B. cereus sensu lato* |  | 2003/43 |  | Wageningen Plant Research |
| *B. cereus sensu lato* |  | 2003/65 |  | Wageningen Plant Research |
| *B. weihenstephanensis* |  | MC118 |  | Aarhus University |
| *B. weihenstephanensis* |  | MC67 |  | Aarhus University |
| *B. mycoides* |  | 2003/84 |  | Wageningen Plant Research |

The *B. thuringiensis* isolates used as biocontrol agents were kindly provided by Valent Biosciences (Libertyville (IL), USA), Certis USA (Columbia (MD), USA) and Probelte (Murcia, Spain). Other strains were provided by the Dutch National Institute for Public Health and the Environment (RIVM)(Bilthoven, the Netherlands), Aarhus University (Aarhus, Denmark) and Wageningen Plant Research (Wageningen, the Netherlands)
