# Supplementary material for: Identification and characterization of Bacillus thuringiensis and other Bacillus cereus group isolates from spinach by whole genome sequencing
Source: Front Microbiol. 2022 Nov 30;13:1030921. doi: 10.3389/fmicb.2022.1030921 (PMC9771606; doi:10.3389/fmicb.2022.1030921)
Supplement: Supplementary file 3 [file Table_3.docx]

Table S3. gBlock sequences (amplicon in black, extended in red)

| **Target** | **Sequence** |
| --- | --- |
| *cry1AaAb* | TATGAGGGTACGTACACTTCTCGTAATCGAGGATATGACGGAGCCTATGAAAGCAATTCTTCTGTACCAGCTGATTATGCATCAGCCTATGAAGAAAAAGCATATACAGATGGACGAAGAGACAATCCTTGT |
| *cry2Aa* | TTAATTATAGCGGAGGAGTTTCATCTGGTCTCATAGGGGCGACTAATCTCAATCACAACTTTAATTGCAGCACGGTCCTCCCTCCTTTATCAACACCATTTGTTAGAAGTTGGCTGGATTCAGGTACAGATCGAGAGG |
| *cry1D* | GCTTATTATGCTGGAACGTGAAAGGGCATGTAGAGGTAGAAGAACAAAACAATCACCGTTCAGTCCTGGTTATCCCAGAATGGGAGGCAGAAGTGTCACAAGAGGTTCGTGTCTGTCCAGGTCGTGGCT |
