# Supplementary material for: Identification and characterization of Bacillus thuringiensis and other Bacillus cereus group isolates from spinach by whole genome sequencing
Source: Front Microbiol. 2022 Nov 30;13:1030921. doi: 10.3389/fmicb.2022.1030921 (PMC9771606; doi:10.3389/fmicb.2022.1030921)
Supplement: Supplementary file 7 [file Table_7.DOCX]

**Table S7 The pairwise wgSNP difference matrix of 10 Bt isolates in ST 15 using the assembly of xentari as the reference genome.**

|  | B1 | B16 | B29 | B30 | B31 | B34 | B4 | B6 | B7 | xentari |
| --- | --- | --- | --- | --- | --- | --- | --- | --- | --- | --- |
| B1 | 0 | 4 | 3 | 4 | 6 | 3 | 2 | 5 | 4 | 2 |
| B16 | 4 | 0 | 2 | 2 | 6 | 1 | 1 | 3 | 4 | 2 |
| B29 | 3 | 2 | 0 | 3 | 5 | 2 | 1 | 4 | 3 | 1 |
| B30 | 4 | 2 | 3 | 0 | 6 | 1 | 1 | 5 | 4 | 2 |
| B31 | 6 | 6 | 5 | 6 | 0 | 5 | 4 | 7 | 6 | 4 |
| B34 | 3 | 1 | 2 | 1 | 5 | 0 | 0 | 4 | 3 | 1 |
| B4 | 2 | 1 | 1 | 1 | 4 | 0 | 0 | 3 | 2 | 0 |
| B6 | 5 | 3 | 4 | 5 | 7 | 4 | 3 | 0 | 5 | 3 |
| B7 | 4 | 4 | 3 | 4 | 6 | 3 | 2 | 5 | 0 | 2 |
| xentari | 2 | 2 | 1 | 2 | 4 | 1 | 0 | 3 | 2 | 0 |
