# Supplementary material for: Identification and characterization of Bacillus thuringiensis and other Bacillus cereus group isolates from spinach by whole genome sequencing
Source: Front Microbiol. 2022 Nov 30;13:1030921. doi: 10.3389/fmicb.2022.1030921 (PMC9771606; doi:10.3389/fmicb.2022.1030921)
Supplement: Supplementary file 8 [file Table_8.DOCX]

**Table S8 The pairwise wgSNP difference matrix of three Bt isolates in ST 8 using** **the assembly of delfin as the reference genome.**

|  | B13 | B33 | delfin |
| --- | --- | --- | --- |
| B13 | 0 | 0 | 8 |
| B33 | 0 | 0 | 12 |
| delfin | 8 | 12 | 0 |
