# Supplementary material for: Identification and characterization of Bacillus thuringiensis and other Bacillus cereus group isolates from spinach by whole genome sequencing
Source: Front Microbiol. 2022 Nov 30;13:1030921. doi: 10.3389/fmicb.2022.1030921 (PMC9771606; doi:10.3389/fmicb.2022.1030921)
Supplement: Supplementary file 10 [file Image_1.pdf]

Supplementary Material

1 Supplementary Figure

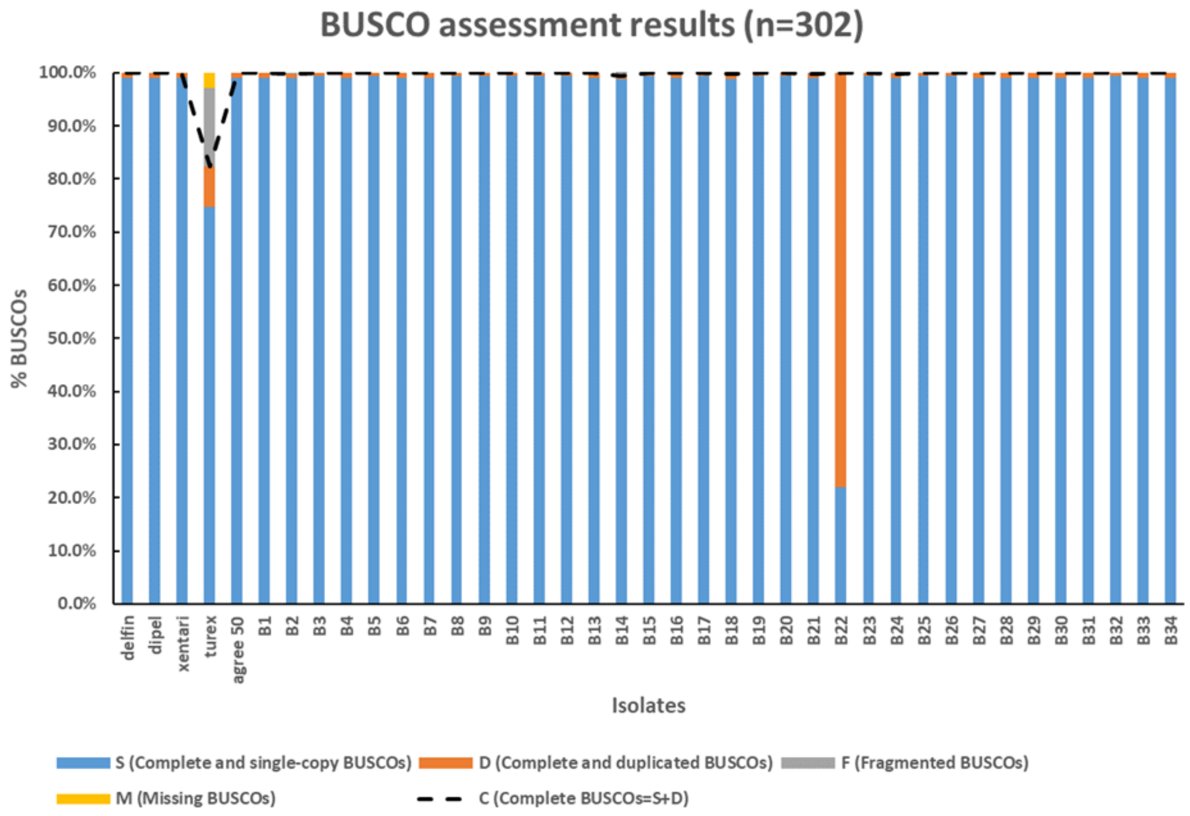

Supplementary Figure 1. Benchmarking Universal Single-Copy Orthologs (BUSCO) assessment of the assemblies from tested isolates. ‘n=302’ represents total BUSCO groups searched from dataset bacilli_odb10.
